# Is the transdermal fentanyl patch an efficient way to achieve acute postoperative pain control? A randomized controlled trial

**DOI:** 10.1097/MD.0000000000013768

**Published:** 2018-12-21

**Authors:** Ji Su Jang, Sung Mi Hwang, Youngsuk Kwon, Hyunjin Tark, Young Joon Kim, Byoung Yoon Ryu, Jae Jun Lee

**Affiliations:** a Department of Anesthesiology and Pain medicine, Hallym University Chuncheon Sacred Heart Hospital; b Department of Anesthesiology and Pain Medicine, College of Medicine, Kangwon National University; c Department of General Surgery, Hallym University Chuncheon Sacred Heart Hospital, Chuncheon, South Korea.

**Keywords:** fentanyl concentration, postoperative pain, transdermal fentanyl patch

## Abstract

**Backgroupd:**

This study investigated the plasma fentanyl concentration and efficacy of transdermal fentanyl patch (TFP) (25 μg/h) in the management of acute postoperative pain.

**Methods:**

Patients undergoing laparoscopic cholecystectomy were randomly allocated to 2 groups. The TFP group (n = 30) received a single TFP 25 μg/ h to the anterior chest wall 14 h before operation. The IV group (n = 30) received a placebo patch. After the operation, intravenous fentanyl infusion (25 μg/h) was begun with loading dose 25 μg in the IV group and only normal saline in the TFP group. Plasma fentanyl levels were measured at admission, 1, 6, 12, 24, and 48 h postoperatively. Pain severity and adverse effects were evaluated too.

**Results:**

The fentanyl level peaked 1 h after operation in the TFP group (3.27 ± 0.34 ng/mL) and 24 h postoperatively in the IV group (2.9 ± 0.42 ng/mL). Pain scores and the use of rescue analgesics were not significantly different between 2 groups. Respiratory depression was not happened in both groups.

**Conclusions:**

The TFP (25 μg/h) affixed 14 h before surgery reached a higher constant concentration than the same dose setting of a constant IV infusion of fentanyl after surgery. Although the concentration of fentanyl was higher than those of previous researches, there was no respiratory depression. But, there was no advantage of reducing pain score and the use of rescue analgesics. Clinical trial registration: (available at: http://cris.nih.go.kr, KCT0002221).

## 1 Introduction

Postoperative pain management is a major concern in surgical patients and inadequate postoperative pain relief is their most frequent complaint. The optimal analgesic modality should be effective, easy to use, safe, and economical. However, adequate pain control without the adverse effects remains challenging. Postoperative pain is commonly managed by intravenous patient-controlled analgesia (IV-PCA) using opioids, such as fentanyl, morphine, or meperidine.^[1]^ The transdermal fentanyl patch (TFP) has been used for chronic pain in patients with cancer since its introduction in 1987.^[2]^ Although the efficacy and safety of TFP for acute postoperative pain management have been investigated, studies measuring plasma fentanyl concentration were too old and recent studies assessed the TFP for postoperative pain control relied on a pain score, rescue analgesic consumption, and the incidence of adverse effects.^[3–9]^

The aim of this study was to assess the plasma fetanayl concentration after TFP (25 μg/h) applied and the efficacy of a TFP applied 14 h before surgery compared to that of an IV constant fentanyl infusion in the management of postoperative pain by pain scores and adverse effects after laparoscopic cholecystectomy.

## 2 Methods

### 2.1 Patient selection

This prospective study was approved by the Institutional Review Board of Hallym University Chuncheon Sacred Heart Hospital (No. 2015-93). Written informed consent was obtained from all enrolled patients and conducted according to the Declaration of Helsinki standard. The protocol of this clinical trial was registered on the Clinical Information Service (available at: http://cris.nih.go.kr, KCT 0002221). The inclusion criteria were patients from 20 to 80 years of age who were scheduled for elective laparoscopic cholecystectomy under general anesthesia with American Society of Anesthesiologists (ASA) physical status I–II from October in 2015 to May in 2016. Patients with a history of allergy to fentanyl, major organ disease, a history of alcoholism or drug abuse, obstructive sleep apnea, fever, or obesity (body mass index > 35 kg/m^2^) were excluded.

### 2.2 Study design and anesthesia

The patients admitted 1 day before surgery, at which time they were randomized into 2 groups using a computer-generated, permuted-block schedule (block size = 4). The assignments were concealed in opaque envelopes and opened immediately before choosing study drugs by a nurse who was blinded to this study and was responsible for preparing the study drugs. Patients in the TFP group received a single TFP (Duragesic matrix fentanyl patch, release rate of 25 μg/h; Janssen Pharmaceutica, Beerse, Belgium) affixed to the anterior chest wall 14 h before surgery. The IV group received a placebo patch. Pre-anesthetic medication was not administered to patients in either group. Laparoscopic cholecystectomy was performed by one surgeon. Anesthesia was induced with 1.5-2.5 mg/kg propofol and 0.8 mg/kg rocuronium. After tracheal intubation, anesthesia was maintained with desflurane-O_2_ (1.5 L/min) and N_2_O (1.5 L/min). Ramosetron (0.3 mg) was administered approximately 15 min before the expected end of operation. After the operation, the patients were transferred to the post-anesthetic care unit (PACU), where a disposable balloon pump for constant infusion of fentanyl (25 μg/h) was started without a loading dose in the IV group. Only normal saline was administered to the TFP group. The TFP and the pump were removed 48 h after the operation.

### 2.3 Measuring of plasma fentanyl concentration

Fentanyl concentrations were measured in blood taken from the peripheral vein at admission (baseline) and 1, 6, 12, 24, and 48 h postoperatively. Venous blood was centrifuged at 3000 rpm for 10 min, and the plasma was frozen within 30 min at -70°C until used in the assays, which were performed at the end of the study. Fentanyl concentrations were measured using commercially available enzyme-linked immunosorbent assay (ELISA) kits (Fentanyl ELISA KIT; Mybiosource, San Diego, CA). The detection limit of the assay was 0.02 ng/mL.

### 2.4 Adverse effects and postoperative pain score

Adverse effects, including nausea, vomiting, dizziness, itching, and respiratory depression (oxygen saturation < 90%), were monitored continuously from attachment of the TFP or placebo patch to 48 h postoperatively. Respiratory depression was monitored by pulse oximetry. Pain severity at cough was evaluated 1, 6, 24, and 48 h postoperatively. Pain was assessed according to an 11-point numeric rating scale (NRS), which ranged from 0 (no pain) to 10 (most severe pain imaginable). When the NRS score was ≥ 4, 30 mg of IV ketorolac was given in the PACU and 50 mg of tramadol was administered in the ward.

### 2.5 Statistical analysis

Sample size was calculated using a power analysis (α = 0.05; power = 0.9) based on our preliminary study. The mean concentrations of fentanyl 12 h postoperatively were 3.181 ng/mL in the TFP group and 2.719 ng/mL in the IV group. The standard deviation (SD) was 0.510. Thus, 27 patients were required in each group, and 68 patients were recruited to take into account an estimated dropout rate of 20%. The SPSS 24.0 (SPSS Inc., Chicago, IL) was used for the statistical analysis. Student's *t* test was performed for continuous normally distributed variables. Fentanyl concentrations at each time point were compared between the 2 groups using a *t* test or the Mann–Whitney *U* test. Categorical variables, including incidence of adverse effects, and consumption of rescue analgesics were analyzed using the Chi-square test or Fisher's exact test, as appropriate. The *P*-values < .05 were considered significant.

## 3 Results

**Figure 1:**
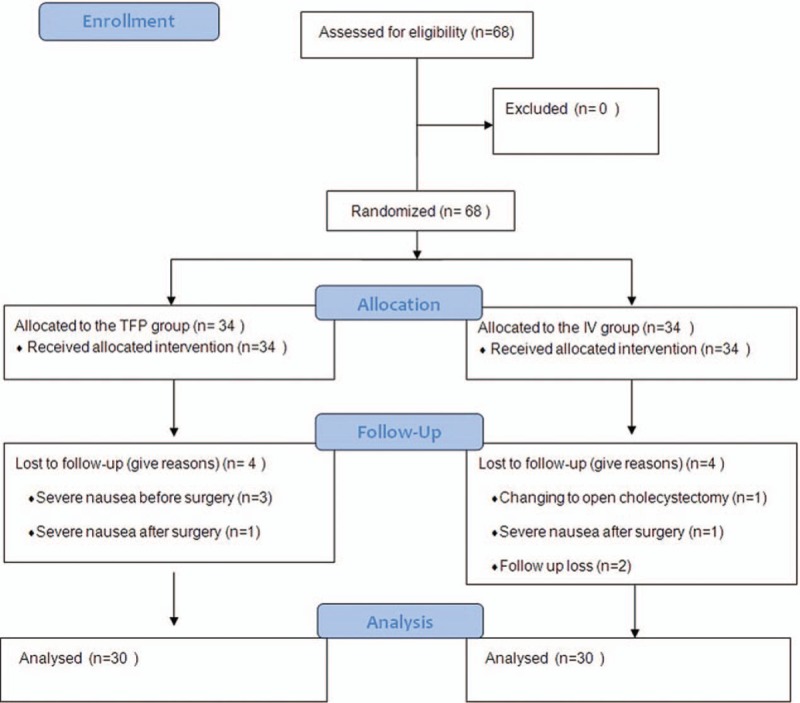
Flow chart of patient enrollment.

Sixty-eight patients who underwent laparoscopic cholecystectomy under general anesthesia were allocated to the TFP and IV groups. Three patients in the TFP group were excluded before surgery because they complained of nausea and therefore declined further participation. One patient in the IV group was excluded due to the need for an open cholecystectomy, which was determined intraoperatively. One patient in each group was excluded after surgery due to nausea. Two patients in the IV group were excluded because their data were lost. Thus, 60 patients were included in the final analysis (Fig. 1).

**Figure 2:**
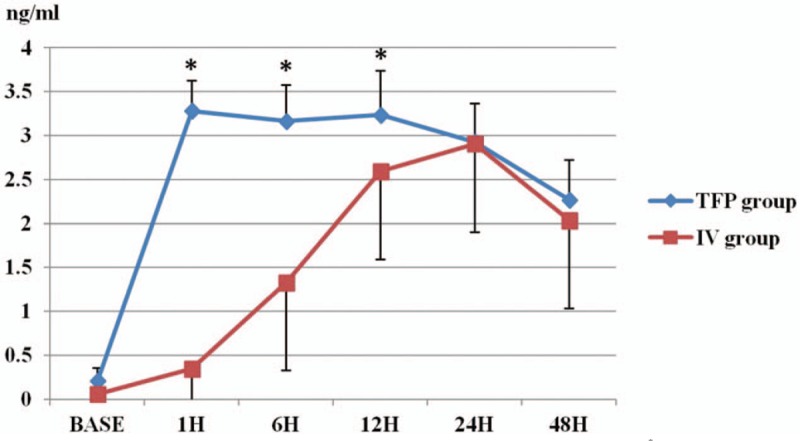
The concentration of plasma fentanyl at admission (Base) and 1–48 h postoperatively. ^*∗*^*P* < .01.

Table 1 shows the demographic data of the patients. Plasma fentanyl level peaked 1 h after the operation in the TFP group (3.27 ± 0.34 ng/mL) and 24 h postoperatively in the IV group (2.9 ± 0.42 ng/mL). Significant differences in the fentanyl level were detected 1, 6, and 12 h after surgery (*P* < .01, Fig. 2). Postoperative pain scores and use of rescue analgesics were not significantly different between the 2 groups (Table 2). Table 3 shows the incidence of adverse effects. Respiratory depression and itching did not occur in any patient. The incidence of nausea during the postoperative period was 7 in the TFP group and 5 in the IV group. Nausea occurred in 10 patients in the TFP group during the entire study period. However, there was no significant difference between the 2 groups.

**Table 1:**
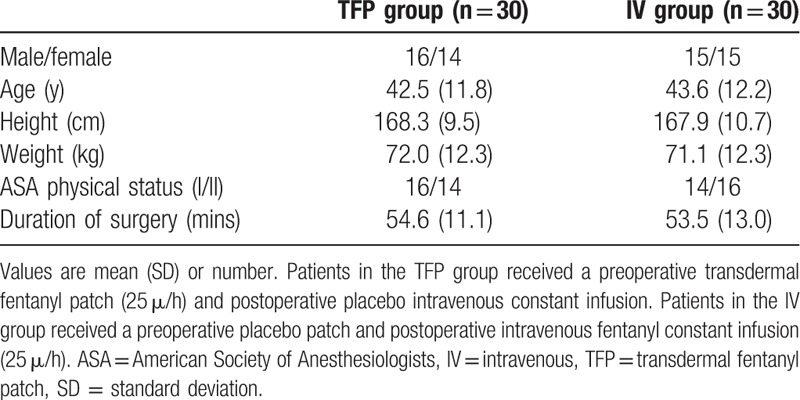
Patient demographics and clinical characteristics.

**Table 2:**
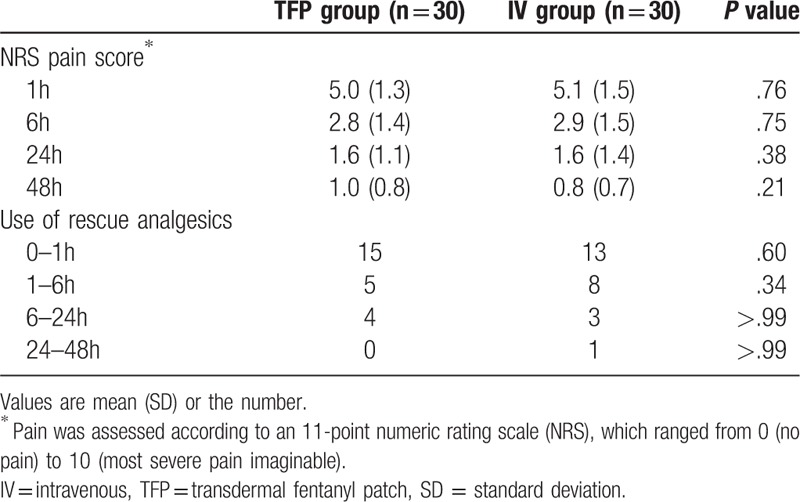
Postoperative pain score and the use of rescue analgesics. Values are mean (SD) or the number.

**Table 3:**
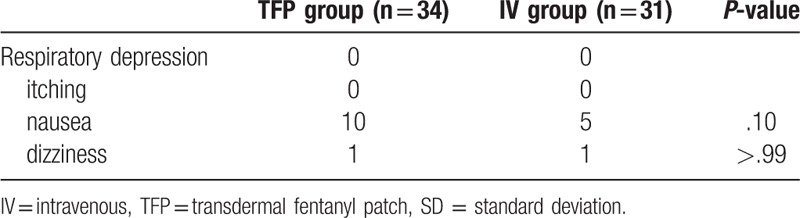
Incidence of adverse effects during whole period.

## 4 Discussion

In this study, the peak fentanyl concentration (3.27 ± 0.34 ng/mL) in the TFP group occurred 1 h postoperatively, which was approximately 16 h after the patch had been applied. The concentration was relatively constant from that time until the patch was removed. This time pattern is similar to those in other studies, but the constant concentration was higher than in previous studies. In 1988, Holley and van Steennis reported that the steady-state serum concentration in patients with a 100 μg/h TFP was 2.15 ± 0.93 ng/mL.^[10]^ In a study reported in 1989, the concentration of fentanyl 24 h after applying 75 μg/h TFP was 1.46 ± 0.97 ng/mL.^[3]^ In another study in 1995, fentanyl concentration showed a wide range (0.85–2.95 ng/mL) and respiratory problems developed in 1 patient whose concentration of fentanyl was 1.79 ng/mL.^[6]^

In a study that evaluated the fentanyl concentration using a 100 μg/h transdermal fentanyl delivery system, serum fentanyl concentration increased gradually during the 1st 14 h after attaching the patch, and was relatively constant from 14 to 24 h (1.8 ± 0.8 ng/mL).^[11]^ However, most of the studies measuring fentanyl concentrations were performed too long ago. New researches for the TFP need to be performed with the remarkable laboratory technology.

Even with the lower-dose fentanyl patch used in the present study, higher fentanyl concentrations were obtained. However, significant adverse effects such as respiratory depression did not occur. When applied 50 and 75 μg/h TFP 2 h before abdominal hysterectomy, apenic episodes, incidence of slow respiratory rate, and increased requirement for oxygen supplementation were increased between 5 and 36 h after surgery. The fentanyl concentration was various when the patients were withdrawal due to respiratory depression (0.93–2.23 ng/mL).^[12]^ The previously reported fentanyl concentrations associated with 50% depression of CO_2_ responsiveness were in the range of 1.5–3.0 ng/mL and hypoventilation occurs in 15% of patients at levels > 1.75 ng/mL.^[13]^ In this study, the peak fentanyl concentration in the IV group was 2.9 ± 0.44 ng/mL 24 h postoperatively, followed by a decrease to 2.0 ± 0.89 ng/mL 48 h postoperatively. Because the aim of this study was comparison of the fentanyl concentration between TFP (25 μg/h) and same dose of IV-constant infusion (25 μg/h), the loading dose was not given in IV- group. The loading dose might be essential for acute postoperative pain control. If more blood samples for fentanyl concentration had been taken between 12 and 24 h postoperatively, it would have been more accurate to compare the plateau state time between the 2 groups.

This concentration was also higher than previously reported levels and showed a different pattern of change. Gourlay et al reported the fentanyl blood concentration-analgesic response relationship during the treatment of postoperative pain in 1988.^[14]^ They estimated the minimum effective fentanyl concentration using an IV-PCA set with a basal infusion rate of 20 μg/h and a bolus “demand” dose of 20 μg. The mean minimum effective concentration (MEC) was 0.63 ± 0.25 ng/mL (range, 0.23–1.18). The MEC in the study by Lehmann et al was 1.35 ± 0.86 ng/mL (range, 0.2–8.0).^[15]^ In another study, mean plasma fentanyl concentrations were 0.51 ± 0.19, 1.42 ± 0.14, and 1.90 ± 0.3 ng/mL after continuous IV infusion of 25, 100, and 125 μg/h fentanyl, respectively, and the authors proposed that the 100 and 125 μg/h dose rates produced significant analgesic efficacy.^[10]^

The TFPs are widely used to control chronic and cancer pain.^[2]^ However investigations about the efficacy of TFPs for acute postoperative pain management have still been reported.^[3–9]^ They proposed the efficacy of TFPs by showing a comparison of postoperative pain score, the use of rescue analgesics, and the incidence of adverse effects. Those authors demonstrated that postoperative pain control can be achieved with TFPs at dose rates of 12–50 μg/h without severe adverse effects.^[10,11,13]^ In present study, though higher fentanyl concentration in TFP group than the IV group, there was no significant difference in pain score and use of rescue analgesics.

The TFPs have several advantages over IV-PCA: they are cheaper, carry a lower risk of infection, have a pre-emptive analgesic effect, and are better tolerated by the patient because they do not require IV access. They also avoid the inconvenience of wearing a bulky PCA pump, which limits patient mobility, particularly during the postoperative period. Moreover, unlike IV-PCA, there is no risk of a program error that could lead to death.^[12,16,17]^ In addition, low-dose TFPs can be effective by providing a background of analgesia, which may be helpful in the management of acute postoperative pain.^[4,18,19]^ Another advantage is the relatively smooth pharmacokinetic curve of fentanyl concentration. The mean curve of fentanyl concentration is flat over the period after reaching steady state.^[2]^ So, Minville V et al proposed that the TFP is an alternative for postoperative analgesia because it provides constant analgesia without waiting for the pain to increase.^[16]^

However, some patients suffered severe nausea before surgery and dropped out of this study. It is undesirable that patients suffer adverse effects due to treatment for pain that has not yet occurred. Even removing the patch does not eliminate the symptoms immediately. In addition, delayed onset, large inter-individual variability in pharmacokinetics, and the inability to adjust the dose during the period of application are disadvantages of transdermal patches.^[9,18,20,21]^ The newly developed patient-controlled ionotrophic transdermal fentanyl delivery system is also expensive.

Thickness and temperature in the skin site of application can alter transdermal fentanyl bioavailability and blood flow to and from the site. Application of a TFP to broken skin can cause to increase of blood fentanyl concentration and skin temperature elevation enhances the absorption of fentanyl.^[22]^ However, the chest area is acceptable site for transdermal patch application and blood flow of the skin site has little effect on the systemic drug absorption under normal physiologic condition.^[16,23]^

The variability in pharmacokinetics, patient-dependent risk factors, and multimodal management of acute postoperative pain must be considered to manage acute postoperative pain effectively.^[6,24,25]^ Merivirta R et al reported that a patch delivering fentanyl 12 μg/h did not reduce the need for rescue analgesics or pain score for postoperative pain management.^[8]^ Lehmann KA et al also recommended the TFP (75 μg/h) as a basis for postoperative pain relief although additional doses of analgesics were required.^[4]^

There are some limitations in this study. First, the sample size of this study was limited, so we cannot be assured of the safety of the TFP. Larger sample sizes are needed to confirm the adverse effects of the TFP on postoperative pain management. Second, we used a fixed dose of TFP or continuous fentanyl infusion regardless of patient body weight. This may affect the pain score and the consumption of rescue analgesics. Third, if the measurement for fentanyl concentration had been performed between 12and 24 h postoperatively, the time of reaching steady state of fentanyl concentration in IV-group would have been more accurate and easier to compare the peak concentration time between 2 groups. Forth, we did not follow the fentanyl levels, the postoperative pain scores, or the adverse effects after removing the patches 48 h after surgery. The fentanyl concentration should still be high enough to be monitored 48 h postoperatively. Fifth, we did not consider the blood pressure during the study period. The blood pressure can affect the pharmacokinetics of the TFP. Sixth, when it comes to the efficacy of pain control, the results of this study are unlikely to be applied to all types of surgery.

## 5 Conclusion

In summary, the TFP (25 μg/h) affixed 14 h before surgery reached a higher constant concentration than the same dose setting of a constant IV infusion of fentanyl after surgery. Although the concentration of fentanyl was higher than those of previous researches, there was no respiratory depression. But, there was no advantage of reducing pain score and the use of rescue analgesics.

## Author contributions

Sung Mi Hwang and Jae Jun Lee designed the research, performed experiments, analyzed and interpreted data, and wrote the manuscript. Jisu Jang and Youngsuk Kwon assisted with analyzing data and writing the manuscript. Jisu Jang, Hyunjin Tark and Young Joon Kim performed experiments, and assisted with taking the blood samples. Byoung Yoon Ryu performed the operations.

Sung Mi Hwang and Jae Jun Lee and Jisu Jang edited the manuscript.

**Conceptualization:** Sung Mi Hwang, Jae Jun Lee.

**Data curation:** Jisu Jang, Sung Mi Hwang, Youngsuk Kwon, Hyunjin Tark, Young Joon Kim, Jae Jun Lee.

**Formal analysis:** Jisu Jang, Sung Mi Hwang, Youngsuk Kwon, Jae Jun Lee.

**Investigation:** Hyunjin Tark.

**Methodology:** Young Joon Kim, Byoung Yoon Ryu.

**Writing – original draft:** Jisu Jang, Sung Mi Hwang, Youngsuk Kwon, Jae Jun Lee.

**Writing – review & editing:** Sung Mi Hwang, Jae Jun Lee.

Sung Mi Hwang orcid: 0000-0001-6035-786X.
